# Response to letter regarding: development of plasma ghrelin level as a novel marker for gastric mucosal atrophy after *Helicobacter pylori* eradication

**DOI:** 10.1080/07853890.2022.2053570

**Published:** 2022-03-26

**Authors:** Hideki Mori, Juntaro Matsuzaki, Hidekazu Suzuki

**Affiliations:** Translational Research Center for Gastrointestinal Diseases (TARGID), University of Leuven, Leuven, Belgium; Division of Gastroenterology and Hepatology, Department of Internal Medicine, Keio University School of Medicine, Tokyo, Japan; Division of Pharmacotherapeutics, Keio University Faculty of Pharmacy, Tokyo, Japan; Division of Gastroenterology and Hepatology, Department of Internal Medicine, Tokai University School of Medicine, Isehara, Japan

We appreciate the interest of Yang et al. [1] in our paper “Development of plasma ghrelin level as a novel marker for gastric mucosal atrophy after *Helicobacter pylori* eradication” [2]. First, they are concerned that plasma ghrelin levels cannot assess antral-predominant gastritis. The aim of this study is to develop a non-invasive, endoscope-free marker of gastric mucosal atrophy. With this assumption, the risk of gastric cancer increases in proportion to the development of endoscopic gastric mucosal atrophy [3]. If the risk of gastric cancer is taken into account, it is of great importance to evaluate open gastric mucosal atrophy. Furthermore, in combination with the *H. pylori* test, it is possible to fully assess the future risk of gastric cancer in a non-invasive way.

They also suggested the loss of oxyntic glands can be different in the same range of open-type atrophic gastritis. We agree with them on this point. Furthermore, there is no way in the world to accurately quantify the loss of oxyntic glands. Since histological intestinal metaplasia usually occurs heterogeneously [4], histological assessment of intestinal metaplasia by the Operative Link on Gastric Intestinal Metaplasia Assessment (OLGIM) may vary depending on the sampling site. However, the OLGIM has already been shown to be useful in assessing the risk of gastric cancer [5] and even taking into account the uncertainty of the point biopsy, it can be inferred that the OLGIM reflects the loss of oxyntic glands. Moreover, it has been proven that the OLGIM stage III/IV and open-typed atrophic gastritis (Kimura-Takemoto) had a strong association [6]. Although the true association with the loss of oxyntic glands is not known, it is at least a useful finding that plasma ghrelin levels in our study are associated with both endoscopic gastritis and the extent of histological intestinal metaplasia by OLGIM.

As they point out, hormone secretion is regulated by a feedback system, but it is often the case that the steady-state is altered by specific conditions. For example, plasma ghrelin levels are known to vary with bodyweight [7]. In our study, the data set was relatively homogenous in terms of body weight, so there was no obvious effect of body weight on the plasma ghrelin levels. However, in order to use plasma ghrelin levels as a biomarker for gastric mucosal atrophy, a weight-based cut-off value would be required in the future.
